# Energy targeting of abandoned mines to supply greenhouse energy demand in cold climates

**DOI:** 10.1177/17442591241298657

**Published:** 2024-12-15

**Authors:** Hosein Faramarzpour, Christopher Reddick, Mikhail Sorin, Jasmin Raymond, Michel Grégoire

**Affiliations:** 1 Department of Mechanical Engineering, Université de Sherbrooke, Sherbrooke, QC, Canada; 2 Centre—Eau Terre Environnement, Institut national de la recherche scientifique, QC, Canada; 3 Les Serres St-Élie, Sherbrooke, QC, Canada

**Keywords:** Abandoned mine, dynamic pinch analysis, greenhouse climate model, clustering, energy targeting, thermal energy storage, air handling units (AHU), geothermal

## Abstract

The combination of a Solar Assisted Geothermal Heat Pump system (SAGHP) with a multi-zone greenhouse is investigated to take advantage of water flooding in abandoned open pit mines in Canada. The envisioned system includes an Air Handling Unit (AHU), Heat Recovery Ventilation (HRV), daily Thermal Energy Storage (TES), and daily Domestic Hot Water (DHW). The main objective is to satisfy the greenhouse heating, cooling, and dehumidification loads, for the considered application, while minimizing energy consumption. This analysis is conducted using data extracted from a case study of a commercial, multi-zone greenhouse, considering different daily weather conditions throughout a year. To reduce the computation time, a clustering approach based on the K-Means method is applied to obtain a small number of typical weather days. Elbow, Dendrogram, and Silhouette approaches confirmed that it is possible to represent a year as six different Typical Days (TD), which can be further categorized as Heating only (TD1 and TD2), Heating/Cooling (TD3 and TD4), and Cooling only (TD5 and TD6). Dynamic Pinch Approach (DPA) showed a great ability to target the minimum energy consumption and maximize the potential heat recovery for each typical day. The study focuses on energy targeting, with discussion of preliminary design considerations, such as the solar hot water (SHW) system, Thermal Energy Storage (TES), and heat pumping. Results revealed that mine water can significantly improve the energy system efficiency, specifically where heating/cooling or only cooling is dominant (TD3, TD4, TD5, and TD6). For instance, by integrating an AHU with the greenhouse for the TDs where heating/cooling is dominant, 22.5% energy saving is achievable. The incorporation of heat pumping, waste heat recovery, and solar thermal collectors through mixed direct/indirect heat recovery (i.e. via TES) can reduce hot utility usage in the considered application by as much as 40%.

## Introduction

Geothermal energy, as a promising heat source to meet energy demand of greenhouses, has an advantage over other renewables, namely being a baseload energy source (Schiffner et al., 2022). It is, however, disadvantaged by significant up-front capital cost that often makes geothermal projects uneconomical. The price for a conventional ground source heat pump system for a residential building can reach up to 31,000 USD. The capital cost of a GSHP system depends on many parameters such as drilling costs (53 USD/m) as the most expensive section (Hance, 2005), equipment price (1354 USD/kW), and land cost (Aditya et al., 2020). To resolve this issue, various solutions, with focus on reducing the drilling process cost, comprising anywhere from 40% to 95% of the total capital (Caulk and Tomac, 2017), have been proposed. Extracting geothermal energy from abandoned gas and oil well (Bu et al., 2012; Kurnia et al., 2022), abandoned underground mines or open pit lakes through a heat pumping system is a promising approach. Having said that, US government has decided to spends $16 billion to reactivate some of the abandoned mines (Santos et al., 2022). Quebec, as the biggest province and the second most populated province in Canada, produces around 98% of its electricity from renewable resources while the contribution of geothermal resources is less than 1%. Gunawan et al. (2020) proposed utilizing ground source heat pump system for heating residential building in north of Quebec province where temperature can reach to minus 40°C. In this air temperature, the COP of air source heat pump system can drop to 1, while the COP of a ground source heat pump system can be maintained over 3. This interesting feature triggered some research studies in order to evaluate the potential of utilizing GSHP to meet energy demand of greenhouses in circumpolar in Canada, specifically, north of QC. Piché et al. (2020) integrated a GSHP with a greenhouse, while it was taking advantage of a passive and active TES. In Canada over 2262 flooded abandoned mines have been inventoried that approximately have 18,642 TJ energy (Grasby et al., 2011). These abandoned mines can supply warm water with temperature with range between 5°C and 18+°C. So far, more than 20 examples of operational geothermal systems at abandoned mine sites, globally verified (Preene and Younger, 2014), while only 32 countries have an activate geothermal power plant (Gutiérrez-Negrín, 2024).

Different studies have investigated the integration of solar collectors (photovoltaic cells or solar thermal collectors) with a Ground Source Heat Pump (GSHP) system, which is called a Solar Assisted Ground Source Heat Pump (SAGSHP). For example, Izmir University in Turkey was the host of an experimental study that was conducted for the integration of a GSHP with a greenhouse (Ozgener and Hepbasli, 2005). Benli (2011) experimentally studied the integration of a GSHP with a 30 $m^{2}$ Chinese style greenhouse. He also did an economical assessment of the energy system and calculated the system COP. Later, a solar assisted water to water heat pump, which met the energy demand of a greenhouse in Australia, was investigated (Aye et al., 2010). Maranghi et al. (2023) investigated a solar assisted ground source heat pump system without and with energy storage (batteries) in the arctic area of Quebec. They showed that, after 10 years, the designed system can reduce fuel consumption by 38% and 59% without and with batteries, respectively. However, one of the main problems regarding the use of SAGSHPs is the dynamic behavior of the greenhouse’s thermal loads, such as domestic hot water consumption, space heating and cooling, and humidity control. Hence, a heat storage tank is a required part of these systems, the importance of which has not been investigated for greenhouses. A TES can be integrated with energy system in two approaches, short-term (daily), or long-term (monthly or seasonally). In short-term approach a TES is a buffer tank that can stores the redundant hot water during warmer time, and release it during cold time. Hosseinnia et al. (2021) conducted a numerical study in order to quantify the thermal performance of stratified Thermal Energy Storage (TES) tanks for a single-family residential building. Also, they mentioned that utilizing TES would enable the designer to size the HP for constant load operation (Hosseinnia and Sorin, 2022). They applied the Dynamic Pinch Approach (DPA) to determine the minimum energy requirements for an existing building. Yang and Rhee (2013) also designed an energy system for a greenhouse while taking advantage of daily TES. However, this TES captured and stored the air’s surplus thermal energy during the daytime and delivered it for heating during the night. Their results revealed that this method is not, economically, viable. When it comes to a long-term TES, soil and rock beds, boreholes, and open pit lakes are included. The concept of borehole thermal energy storage (BTES), as a seasonal TES, was successfully integrated with a greenhouse (Semple et al., 2017; Vadiee and Martin, 2013; Zhang et al., 2015).

Another promising way to reduce energy consumption in greenhouses is to take advantage of low enthalpy waste energy such as greywater (Manouchehri and Collins, 2016; Mazhar et al., 2018). In a greenhouse, however, the amount of this waste energy is not significant. The amount of heat loss thorough ventilation systems is estimated to be more than 20% in cold climate conditions (Ghaderi et al., 2023). Also, while a high performance residential building has around 1 air change/h (ACH), this value can reach 60 for a commercial greenhouse (Ghaderi et al., 2023). To mitigate this issue, implementing a Heat Recovery Ventilator (HRV) within a greenhouse has been proposed (Cuce and Riffat, 2015). To calculate the contribution of ventilation to greenhouse energy demand in cold climate conditions, De Halleux and Gauthier (1998) studied three different scenarios for dehumidification purposes: no ventilation, hourly ventilation, and proportional ventilation. Based on their results, proportional and hourly ventilation can increase energy consumption by 18.4% and 12.6%, respectively. They stated that for the province of Quebec, installing HRV equipment is logical if the payback period is less than 4 years. Also, Campen et al. (2003) specified that, under cold climate conditions, air-to-air exchangers can significantly reduce the heat loss due to venting-heating, while Rousse et al. (2000) reported 78% sensible heat recovery by HRVs. Maslak and Nimmermark (2017), based on a study in northern Europe, concluded that HRVs can reduce energy consumption around 15%–17% in the months from April to September.

Another challenge for greenhouse energy targeting is the sizing of equipment, such as HP, solar collector, and TES. Generally, the HP can be sized to meet heating demand + DHW, or cooling demand + DHW. If the HP’s condenser is big enough to supply space heating + DHW during the coldest day of the year, the energy system can undoubtedly meet the greenhouse heating demand, however it will be over-sized. Also, the DHW set point temperature and space heating set point temperature should not be equal, since it can significantly reduce the COP of the HP. Given that greenhouse energy requirements can vary widely both during 1 day and throughout the year, computation time and design complexity can be decreased by reducing the typical meteorological year of 365 days into a small number of typical days. Finding some typical days to size the HP is necessary to target minimum energy consumption. Hosseinnia and Sorin (2021, 2022; Faramarzpour et al., 2018) proposed a monthly design strategy based on maximum energy consumption. For their case, they selected 12 typical days for each year (21st of each month), having observed that the greatest energy demand occurred on January 21st. Ghaderi et al. (2023) proposed a clustering method (K-Medoids) to select the typical days, finding 12 upon which to perform DPA. Schütz et al. (2018) did a comparison study on the accuracy of different clustering methods. They concluded that K-Medoids and K-Means, conditionally, can produce more accurate clusters than alternative methods. Also, Arora and Varshney (2016) showed that the K-Medoids method is more accurate and faster than K-Means. However, most of the reviewed papers highlighted that clustering methods are very case sensitive and that a larger number of clusters can improve the model’s accuracy (Domínguez-Muñoz et al., 2011).

The present study introduces a systematic energy targeting strategy based on DPA to find the best way to combine different sources of energy, including abandoned mine geothermal and solar, within a cold climate greenhouse, while simultaneously minimizing energy consumption. The considered application is that of an abandoned open pit mine and a commercial greenhouse. The abandoned mine water resource of the case study is that of King Beaver Mine near Thetford Mines, Quebec, Canada. The reference greenhouse is Les Serres Saint-Élie, in Sherbrooke, Quebec, Canada. The study evaluates integration possibilities for the ventilation waste heat into the HVAC system of a greenhouse, including the evaluation a low enthalpy geothermal heat pump, a heat storage tank, a solar hot water system, and an AHU/HRV unit to meet all heating, cooling, and dehumidification loads. The novelty of this study is the combination of a multi-zone greenhouse with a solar assisted, geothermal heat pump system, connected to an abandoned mine lake, where the energy system is analyzed with “Dynamic Pinch Analysis” to target minimum energy consumption. Also, a solar hot water system (SHW) is optimized and sized by PA for the first time. To the best of the authors’ knowledge, this is the first time that a systematic pinch approach is applied for sizing and energy targeting of a SAGSHP system for a greenhouse.

### System description and problem statement

**Figure 1.**
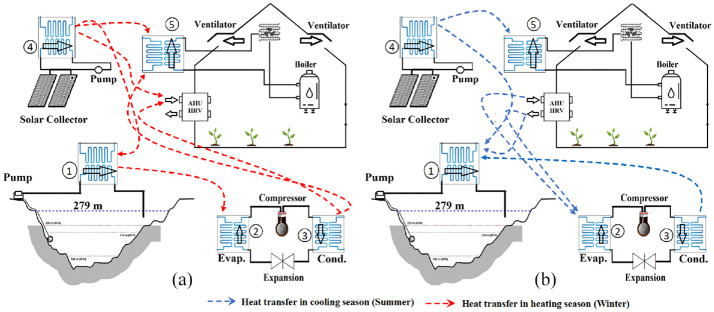
Schematic design of the considered energy system for a case study greenhouse. (a) Heat transfer opportunity in Summer and (b) heat transfer opportunity in Winter.

Figure 1 illustrates the different sources of renewable energy that can be integrated by the current energy system of a case study greenhouse, namely the St. Elie greenhouse. Usually, the energy system of a typical greenhouse is fairly simple. It almost always includes a natural gas or oil-fired boiler, a hot air or water heat distribution system (e.g. a coil), and a roof ventilation system for cooling and dehumidification purposes. However, to maximize the heat recovery potential and incorporate renewable energies (solar and geothermal), adding some equipment is necessary. To reduce the waste energy from roof ventilators, an AHU or HRV is considered. An HRV is equipment that recovers energy (both sensible and latent heat) from air that is being exhausted from a conditioned space (return air) and transfers that energy to incoming fresh air (outdoor air). An AHU is equipment that has a fan and a coil (hot water, chilled water and/or direct expansion coil) that will heat, cool, or simply circulate air to condition a space. An AHU is usually coupled to a chiller, boiler, or external condenser that provides hot or cold water and/or refrigerant flow to the coils.

Also, a solar assisted geothermal heat pump system is sized and integrated to the current energy system of the case study greenhouse. Figure 1 presents some heat transfer possibilities among all heat sources/sinks for the considered energy system. Figure 1(a), shows some possible heat transfer path in summer season, and Figure 1(b), shows those opportunity for winter season. It should be highlighted that all heat transfer paths presented in this figure do not happen simultaneously, and some of them are activated at any given time depending on the system operation mode (i.e. heating/cooling, lake charging/discharging, and ventilation ON/OFF). The optimal interconnection of heat exchanger units creates a HEN that will allow the maximum heat integration potential to be achieved (Ghaderi et al., 2023).

The implementation of the integration concept using pinch analysis comprises two major stages. First, we need to calculate the maximum feasible heat recovery targets, which is the focus of this article. There will be one set of targets for each of the time periods that we choose to evaluate. Second, for each of the time periods, a HEN can be designed to meet the targets as much as possible. Design choices are required to evaluate the various HEN designs and select the optimal. In fact, the major advantage of PA is that it provides the insight for the possible stream matches required for the subsequent HEN design.

## Methodology

**Figure 2.**
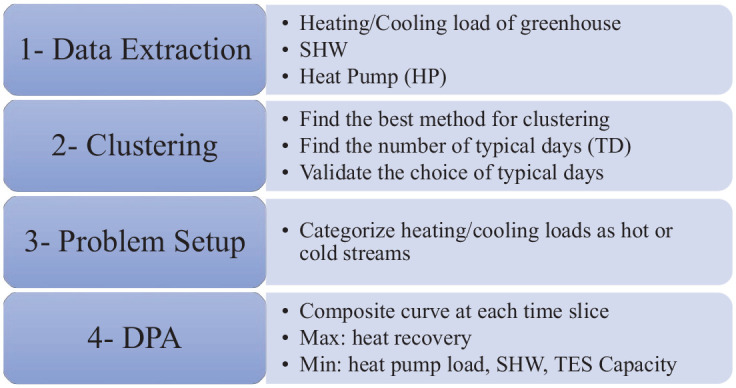
Required steps to accomplish the tasks of this study.

Figure 2, shows the steps to accomplish a heat integration study, with different sources of energy, based on the dynamic pinch approach. By conducting this study, a systematic graphical DPA will provide a model for integrating all potential energy sources into the energy system of a greenhouse, where the maximum heat recovery, the minimum required hot utility, and the optimal equipment sizing can be achieved. The methodology of the current study contains four steps: data extraction, data clustering, problem setup, and dynamic pinch analysis (Ghaderi et al., 2023).

In the data extraction step all heating/cooling loads of the greenhouse are calculated. In the second step, a clustering method (K-means) is applied to the calculated daily heating/cooling loads from the first step, with the goal of finding several typical days that represent 1 year. This greatly reduces calculation time in the subsequent steps. In the third step, all sources of energy are obtained and categorized as hot or cold streams. For example, when it comes to domestic hot water system, the pattern of hot water consumption for the building should be extracted from the literature. HP also can have a hot and cold stream namely condenser and evaporator. The different source of streams in data extraction section (see Data extraction section) are provided. Finally, in the fourth step, the DPA is applied. In this article, the energy targeting is completed within the fourth step, which includes some discussion and preliminary design of specific equipment characteristics (HP capacity, optimal SHW parameters, and volume of TES tanks).

### Data extraction

Data extraction is the step where we need to quantify all dynamic loads of the greenhouse as precisely as possible. Shape, form, orientation, envelope materials, set points (temperature and relative humidity), the outdoor temperature, and the solar irradiance can affect heating/cooling demand of greenhouse. A mathematical energy model developed for a multi-zone greenhouse is applied to calculate heating/cooling and dehumidification loads in the current study. The complete description of both the model and the selected case study are presented in Ghaderi et al. (2023). For convenience some details are also presented here.

#### Heating/cooling load: Data processing (finding typical days)

**Figure 3.**
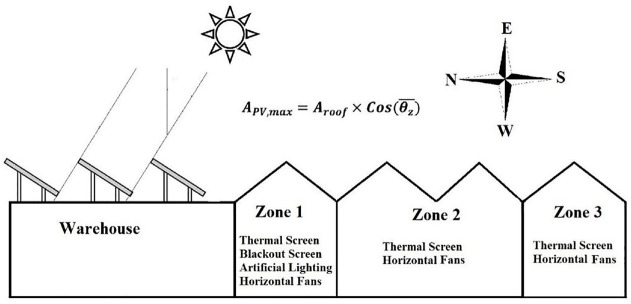
Schematic of case study greenhouse and proposed location of solar collectors.

**Figure 4.**
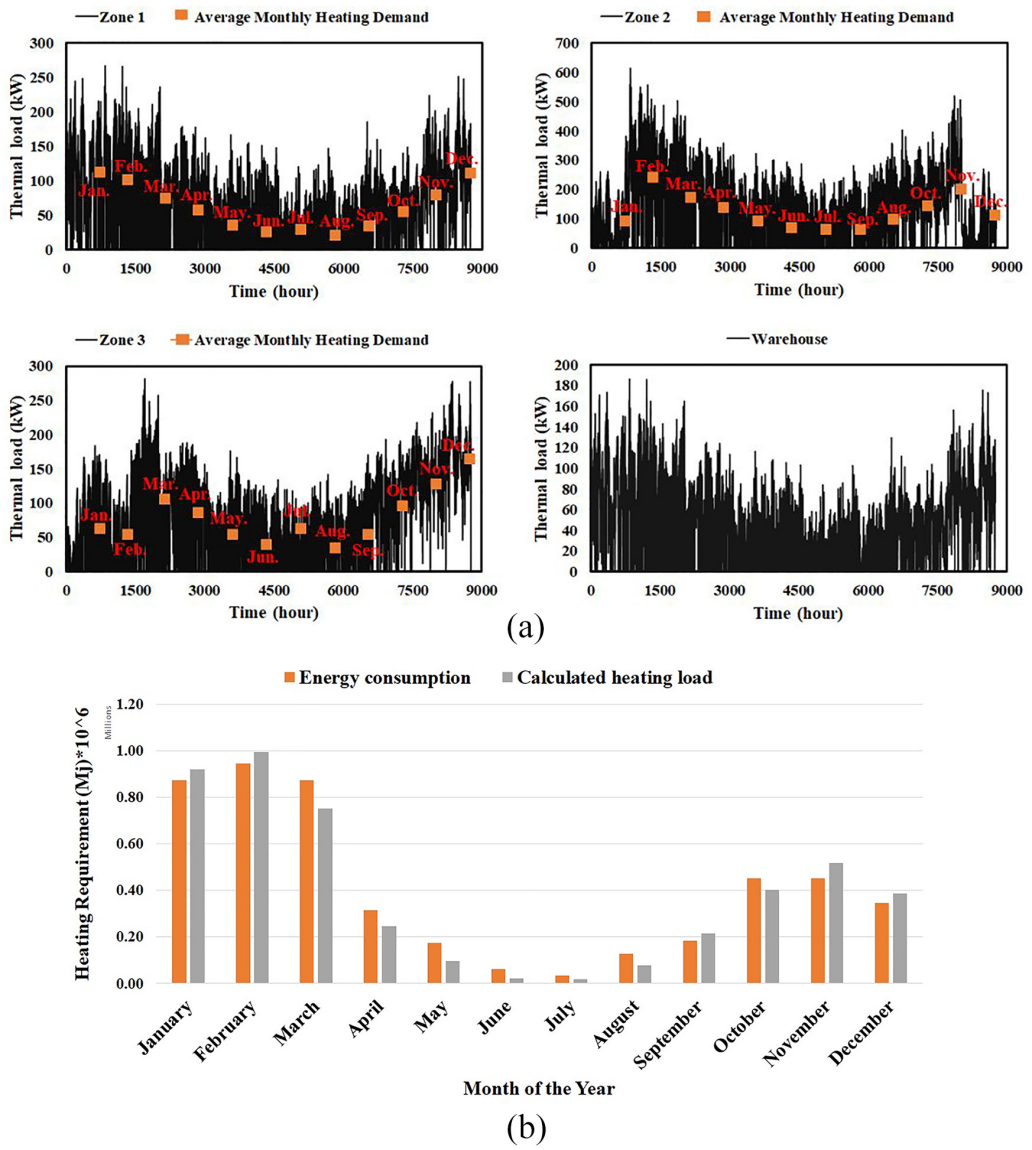
(a) Heating demand of different zones of St. Elie greenhouse and (b) annual variation of the heating requirements in the St. Elie greenhouse versus heating load calculated (Faramarzpour et al., 2024).

To accomplish the current study, a flower greenhouse in Sherbrooke, Quebec, Canada (45.4043°N, 71.8937°W) has been selected. This greenhouse, named “Les Serres St-Élie,” has three different micro-climate zones and a warehouse to support logistical requirements. Also, thermal screens, blackout screens and artificial lighting are employed in the greenhouse to reduce energy consumption and increase productivity. Overall dimensions and micro-climate conditions of the St. Elie greenhouse are illustrated in Faramarzpour et al. (2024). Figure 3, shows a simplified illustration of the case study greenhouse, consisting of three zones, equipment for each zone, and the proposed location for solar collectors for the hot water system. To calculate the energy demand of this greenhouse, a MATLAB-SIMULINK code was developed. It is worthwhile noting that this code can predict the energy demand of the greenhouse in the cold season with accuracy of up to 7%. To calculate the accuracy, Mean Squared Error (MSE) method is utilized. As explained in Faramarzpour et al. (2024) and also presented in Figure 4(b), the accuracy of model, specifically in cold months, is completely acceptable. To do model verification, the calculated energy demand was compared with the actual energy consumption of case study greenhouse. The actual energy consumption of greenhouse was collected from BAS (Building Automation System) and gas bill.

As explained in Faramarzpour et al. (2024), the St Elie greenhouse has three distinct production zones and a warehouse, each with different micro-climate conditions. This fact causes each zone to have a different heating/cooling demand throughout the year, even in the summer season when the solar radiation is at its maximum value. Figure 4 presents the heating load of the three zones and the greenhouse warehouse, as well as the validation of model. The more detail information can be found in Faramarzpour et al. (2024).

In this study, the clustering task was accomplished by two different methods. In the first method (K-Medoids), a developed MATLAB code, previously used by this research group (Ghaderi et al., 2023), was applied to cluster thermal loads of the case study greenhouse. In the second method, the clustering process was done using K-Means. In cases where value distribution is nonuniform, K-Means is recommended (Yarahmadi et al., 2024). Figure 4 shows that the thermal load distribution is quite nonuniform throughout the year. For clustering by K-Means method, a PYTHON code was developed. Although these methods can break up the 365 daily heating/cooling data into small packages, called typical days (TDs), they do not indicate what the optimum number of clusters should be, which is the most challenging part of data clustering.

**Figure 5.**
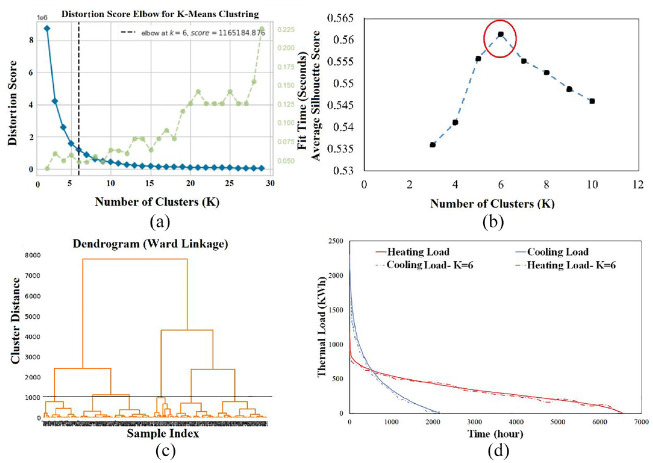
Different approaches to determining the optimum number of clusters: (a) Elbow, (b) Dendrogram, and (c) Silhouette, as well as LDCs, shown in (d).

In this study, to find the optimum number of clusters, three different approaches were employed, namely: Elbow (Figure 5(a)), Silhouette (Figure 5(b)), and Dendrogram (Figure 5(c)). As can be seen in Figure 5, the greenhouse energy demand can be categorized into six different groups. Furthermore, the accuracy of the choice of the optimal number of clusters was examined by introducing Load Distribution Curves (LDC), such as shown in Figure 5(d). LDC graphs plot the hourly heating (red) and cooling (blue) loads of typical days in descending order, for one whole year, and is an effective method to investigate the performance of clustering method. Figure 5(d), depicts the LDCs for a choice of six clusters, as determined by the K-Means method. In comparison, the number of clusters considered by Ghaderi et al. (2023; Faramarzpour, 2024) was 12 using the K-Medoids method, requiring more calculation time.

**Figure 6.**
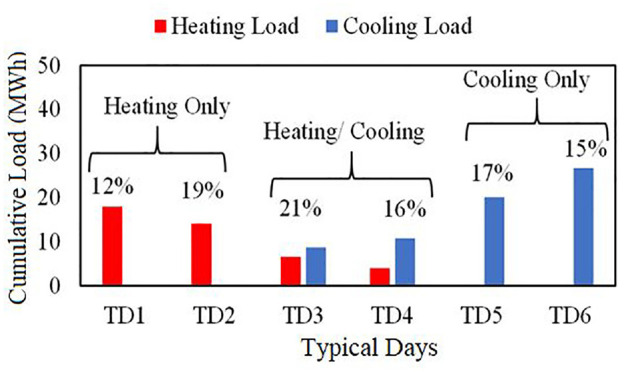
Cumulative demand category and cluster size for typical days.

Figure 6 shows the distribution of the six typical days calculated with the K-means method, representing one whole year. As shown, most of days for this case study need heating load, whether slightly or substantially. The six typical days can be grouped together according to the category of heating and/or cooling demand: Heating Only (TD1 and TD2), Heating/Cooling (TD3 and TD4), or Cooling Only (TD5 and TD6). Once the typical days are chosen, it is possible to appropriately size the heat pump system. The Cumulative demand for each TD is calculated for 24 h of a day. It is the summation of the energy demand in each time interval plus the value of energy demand in the previous time interval (Ghaderi et al., 2023; Hosseinnia and Sorin, 2021, 2022).

#### Geothermal resource: King beaver mine

A Ground Source Heat Pump system (GSHP), in comparison with other heating systems like an Air Source Heat Pump (ASHP), shows the highest COP, especially when the ambient air temperature is equal/less than 0°C. Quebec generally has less than 100 frost-free days annually. Hence, extracting energy from geothermal resources is a promising method in the province of Quebec. In the city of Thetford Mines, Quebec, Canada, the open pit lake of King Beaver mine can potentially supply more than 800 kW of thermal energy for a heat pump system for over 25 years (Comeau et al., 2019). The energetic study of this mine has been conducted by Institut national de la recherche scientifique (INRS). Based on this report, the water temperature in open pit lake of King Beaver mine is constant and around 5°C.

In this study, while the initial extracted water temperature is considered fixed and equal to 5°C, the extracted/injected load is variable and will be calculated based on PDA. This heat source/sink works in two different modes, namely extracting energy from the lake during a greenhouse heating demand, and injecting energy into the lake during a greenhouse cooling demand. The main constraint to set inlet/outlet temperature from this geothermal resource is the freezing point of open pit lake water. Chemical analysis of the lake water shows a high concentration of calcium and other chemical substances. Hence, the freezing temperature of the lake can be considered less than 0°C but to be conservative, this temperature is considered 0°C.

#### Heat pumping

Heat pumping plays an important role in addressing the heating and cooling demands of the case study greenhouse to integrate lower temperature energy sources, such as geothermal or waste heat from ventilation. The main source of energy to meet the energy demand of the greenhouse will be a water-to-water HP. Both Hosseinnia and Sorin (2021) and Ghaderi et al. (2023), as part of their PA analyses, demonstrated the essential function of heat pumping in high efficiency greenhouse energy system design. The following lists the HP assumptions for this study.

1- Environmental concerns: To address this issue refrigerant R466A is selected.
2- Minimum refrigerant pressure: The minimum HP cycle pressure is 620 kPa.
3- Maximum and minimum cycle temperature: In a PA problem the temperature range of different source of energies is important. Hence, the minimum and maximum temperature of whole streams should not cross the evaporator/condenser range. Hence, the assumed temperatures for the evaporator and condenser are −10°C and 55°C, respectively.
4- Maintenance cost: The considered HP in this study works under constant load.
5- COP range: The proper COP range is 2.2 < COP < 5.8 (Schlosser et al., 2019).
6- To increase efficiency and minimize compressor malfunction, 5°C superheating, and supercooling for the evaporator and condenser are assumed, respectively.

**Figure 7.**
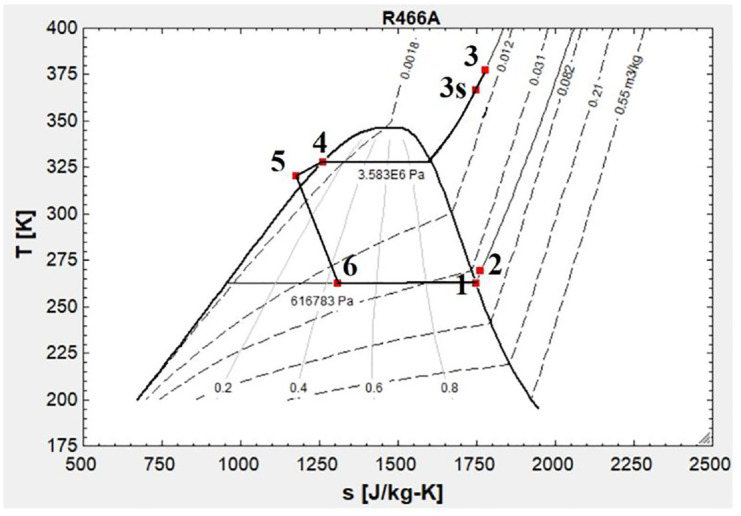
T-s diagram for the designed HP.

Figure 7 shows the T-s (Temperature-Entropy) diagram (R466A) of the considered heat pump system. To facilitate the thermodynamic calculations, EES software (Academic V10.836) was employed. As presented in Figure 7 COP is calculated 3.47.

#### Intermediate heat recovery loops and stratified thermal energy storage tanks

As pointed out by Hosseinnia and Sorin (2022), adding a TES can help to design a HP with constant load, which reduces the size of heat pump, and consequently decrease the capital and operational costs of an energy system. Also, in a practical situation there is always a mismatch between available hot water and energy demands. Furthermore, it is possible an energy system will occasionally face extreme weather conditions. A stratified thermal storage tank (TES) can guarantee the reliability of the energy system in these situations. For these reasons a TES is added to the considered energy system.

#### Air handling unit

**Figure 8.**
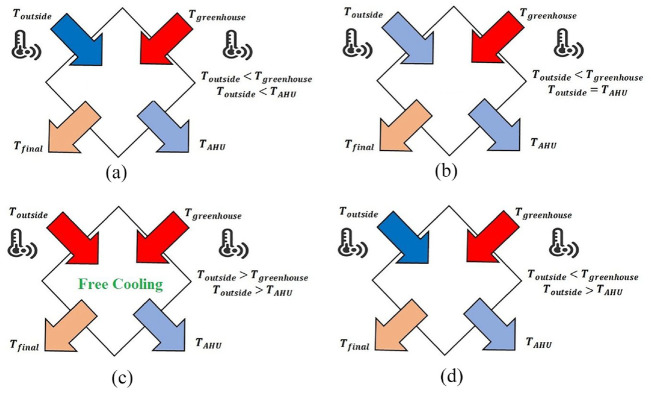
Schematic diagram of AHU streams under inside/outside air temperature.

The main purpose of integrating this equipment within a greenhouse is to take advantage of the exhausted hot and humid air, which mainly occurs during summer or on occasional warm days in cold seasons. An AHU is manufactured from various components, including heat exchangers and cooling equipment. In this study, to calculate $T_{\mathrm{AHU}}$ and also its capacity, the method of Ghaderi et al. (2023) is utilized. The calculation of $T_{\mathrm{AHU}}$ is based on the outside temperature, inside temperature, and inside relative humidity. This means, to determine the hot and cold streams of this equipment, in addition to quantifying the heating/cooling demand of greenhouse, the dehumidification load needs be calculated. Briefly, the calculation process is initiated by importing the greenhouse weather data and heating/cooling demand for a typical day. Then, based on outside air temperature (*T*_*outside*_), the AHU leaving temperature (*T*_*AHU*_), and whether the incoming air needs to be heated or cooled, the required mass flow rate of supply air is determined. For an AHU integrated with the greenhouse, four different operating modes are possible. Just as a sample, Figure 8(a) shows a situation where the greenhouse needs cooling and/or dehumidification, while ambient temperature is less than the greenhouse and AHU temperatures. In this situation, an energy source is needed to increase outside temperature to $T_{\mathrm{AHU}}$ . To do this, the leaving greenhouse air can transfer heat to the incoming supply air. The exact behavior of the AHU should be determined by pinch analysis for each of the possible operating modes.

#### Household hot water consumption

**Figure 9.**
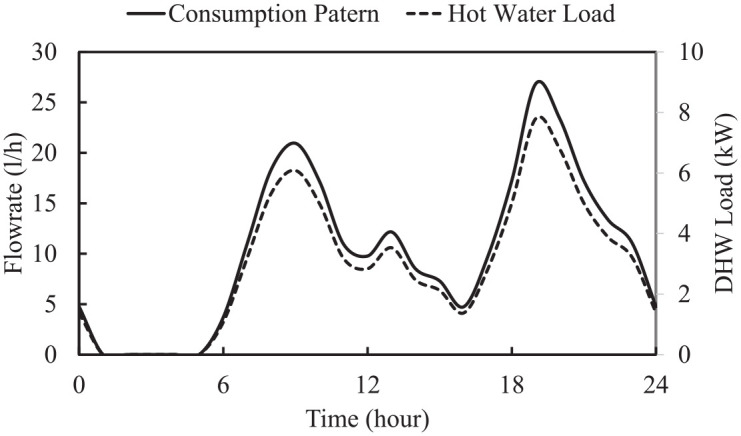
Hot water mass flow rate and its thermal load for the case study greenhouse for TD1.

In addition to heating load, hot water consumption makes a significant contribution to the greenhouse energy requirements. In a residential building, the hot water consumption depends on different parameters, among which the number of residents and the season make the highest contribution. However, there is no published literature that reports the pattern of hot water consumption for a greenhouse. Hence, since the number of staff are almost equal to number of people in a family, in this study the consumption pattern of Canadian family is selected. Based on a review of the literature, the estimation for hot water consumption for a building in North America, specifically Canada, is 249 L/day (Perlman and Mills, 1985). The distribution pattern for this amount of hot water consumption in a day can be calculated based on different methods: Rand, constant, early morning, etc. In this study the Rand profile (Mutch, 1974) is selected. Figure 8 shows the hot water consumption pattern as well as the associated heating to increase its temperature from city water temperature (Table 1) to 55°C. In Figure 9, this information is presented on the right-side vertical axis as the domestic hot water (DHW) load.

**Table 1.** Monthly variation of city water temperature for 1 year in Montreal (Hosseinnia and Sorin, 2022).

Time	January	February	March	April	May	June	July	August	September	October	November	December
Temp. (°C)	5	5	5	8	9	10	10	8	8	8	7	6

#### Solar hot water system

The most important parameters that can affect the performance of the SHW are the time of year and day, the air temperature, and solar collector efficiency. The first step in determining the solar collector duty is to calculate the solar irradiance, G(t) , for each typical day. To read more about the calculation of solar irradiance (W/ $m^{2}$ ) please refer to Faramarzpour et al. (2024). By using the calculated G (W/ $m^{2}$ ) and equation (1), the solar collector duty for 50 $m^{2}$ of flat solar collector can be determined. To select the solar collector area, different criteria are involved, namely energy demand, available installation area, financial issues, etc. However, in this study, the assumed solar collector area is sufficient to meet DHW demand of the greenhouse and cover the warehouse width of 50 m.

(1) $$Q_{\mathrm{SHW}}=A_{c}(\eta_{0}G-a_{1}\left(T_{c}-T_{\mathrm{out}}\right)-a_{2}{\left(T_{c}-T_{\mathrm{out}}\right)}^{2})$$

**Figure 10.**
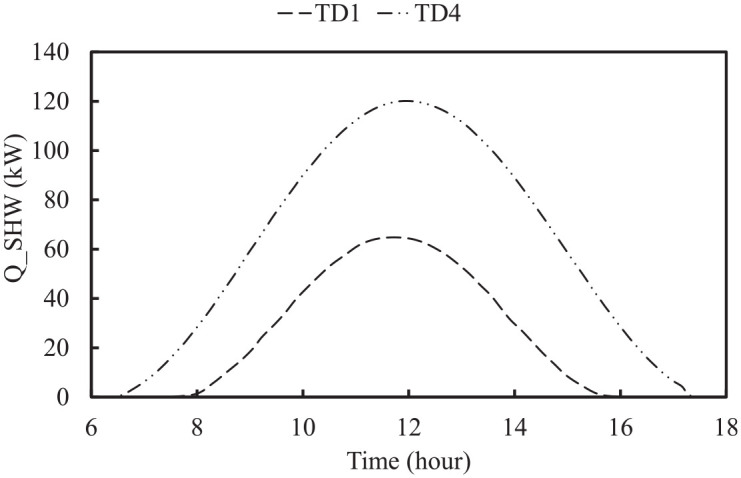
Solar collector duty for two typical days.

Sung et al. (2015) introduced a function to predict the ambient temperature ( $T_{\mathrm{out}}$ ) based on min/max temperature of each day. However, in this study, meteorological TMY2 data were used. In equation (1), the values for $a_{1}$ , $a_{2}$ , and $\eta_{0}$ are taken from Hosseinnia and Sorin (2021). Also, the collector temperature ( $T_{c}$ ) was calculated as ( $T_{\mathrm{in}}+T_{\mathrm{out}}$ )/2. The city water temperature ( $T_{\mathrm{in}}$ ) for each month can be found in Hosseinnia and Sorin (2022), and is presented in Table 1, while $T_{\mathrm{out}}$ is the outlet water temperature from the solar collector. This temperature is set at 65°C. The available solar collector area is directly related to the collector slope and roof area (Figure 3), which in the case study is more than 300 $m^{2}$ . However, in this study only 50 $m^{2}$ of solar thermal collector are assumed to be installed to supply 500 L/day of hot water for the case study greenhouse. The mass flow rate of designed SHW is selected based on number of staff and their schedule in greenhouse study case (Perlman and Mills, 1985). Figure 10 shows the available solar energy in TD1 and TD4.

#### Hydronic floor heating (HFH) and ceiling heating loop (CHL)

The heating system for the St. Elie greenhouse mainly consists of two different mechanisms: floor heating, and ceiling heating. In the floor heating mechanism, the inlet and outlet temperatures are set to 35°C and 30°C respectively. Also, this range for the ceiling heating loop is considered 45°C and 40°C, respectively. The mass flow rate of hot water to serve hydronic floor heating and tubes is calculated based on heating demand in Heating/cooling load- Data Processing (Finding typical days) section. The hot water temperature at the TES tank exit should be regulated by a mixing valve. Also, the HFH temperature is limited to 35°C to prevent discomfort for the staff or damaging the floor.

### Problem setup

The ultimate goal of the problem setup is to fully define the streams to be used in the heat integration. After defining the actual and potential sources/sinks of energy for each typical days (TD1-TD6), it is possible to determine the sets of cold and hot streams. In Table 2, the initial and final (i.e. target) stream temperatures, amount of heat, and type of streams (hot/cold) are defined. The next step is plotting CCs for 24 h for each typical day. In this way it is possible to, first, identify the potential heat recovery resources, and second, determine the required amount of heating/cooling utility for the energy system. Also, DPA helps to find appropriate pairs of hot and cold streams. Table 2 also presents shifted temperatures for the streams. To understand the concept of shifted temperature, Smith (2005) can provide worthwhile information. Here, however, this concept is briefly explained. To obtain the shifted temperature, the initial and final temperature of the hot streams should subtract $\Delta T_{\min}/2$ , while cold streams temperatures are increased by $\Delta T_{\min}/2$ . In this study $\Delta T_{\min}$ is assumed to be 5°C. In this way hot and cold composite curves will touch each other at the pinch point if the CCs are plotted with shifted temperatures.

**Table 2.** Hot and cold streams with supply and target temperatures.

Streams	$T_{\mathrm{Initial}}$ (°C)	$T_{\mathrm{Target}}$ (°C)	$T_{\mathrm{Shifted},\mathrm{Initial}}$ (°C)	$T_{\mathrm{Shifted},\mathrm{Target}}$ (°C)	Q (kW/°C)	Material
Evaporator (C)	−10	−10	−7.5	−7.5	Constant	R466A
Evaporator (superheat) (°C)	−10	−5	−7.5	−2.5	Constant	R466A
Condenser (superheat) (H)	104.35	55	101.85	52.5	Constant	R466A
Condenser (H)	55	55	52.5	52.5	Constant	R466A
Condenser (subcooling) (H)	55	50	52.5	47.5	Constant	R466A
Geothermal water (evaporator) (H)	4.8	0	2.3	−2.5	Constant	Water
Hydronic floor heating (C)	20	30	22.5	32.5	Dynamic	Water
Ceiling heating loop (C)	40	45	42.5	47.5	Dynamic	Water
Supply air (H/C)	$T_{\mathrm{out}}$	$T_{\mathrm{AHU}}$	$T_{\mathrm{out}}$	$T_{\mathrm{AHU},\mathrm{Shifted}}$	Dynamic	Air
Exhaust air (H)	24	$T_{\mathrm{out}}$	21.5	$T_{\mathrm{out},\mathrm{Shifted}}$	Dynamic	Air
SHW (H)	60	40	57.5	37.5	Dynamic	Water
IHRL-H (H)	55	30	52.5	27.5	Dynamic	Water
DHW (C)	$T_{\mathrm{City}\;\mathrm{water}}$	55	7.5	57.5	Dynamic	Water
CL (H)	4.8	12	2.3	9.5	Dynamic	Water

**Figure 11.**
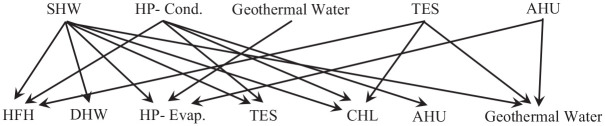
Possible heat exchanges between streams on a typical cold day in winter.

Figure 11 shows possible pairs of hot streams (upper row) and cold streams (lower row) in the considered energy system for the case study greenhouse. On a typical cold winter day, when heating is the greatest demand in the case study greenhouse, the heat pump system extracts energy from the open pit lake of the abandoned mine (King Beaver Mine) to warm the evaporator. On the other hand, the condenser produces hot water and releases it to the TES. The hot water from the TES is later mixed with cold water and supplies the Hydronic Floor Heating (HFH), Ceiling Floor Heating (CHL), and/or Domestic Hot Water (DHW). The maximum target temperatures of HFH, CHL, and DHW are 35°C, 45°C, and 55°C, respectively. It should be highlighted that to supply the required DHW temperature, this study chose using SHW, instead of increasing the heat pump maximum temperature. By doing so, the COP of the heat pump is increased by 8%. The current COP value, as presented in Figure 7, is 3.47. For a cold season, in addition to the heat pump, an AHU is integrated with the energy system to adjust RH and take advantage of heat recovery.

For the hot season, when the greenhouse mostly needs cooling duty, this study chose to make use of a free cooling opportunity, which in addition to being cost efficient, helps to maintain the open pit lake temperature for the cold season. King Beaver Mine can provide an almost unlimited amount of cold water with a temperature less than 5°C (undisturbed temperature), which is an appropriate temperature for a practical cooling tower. Also, this temperature is around 10°C lower than the dew point of air in summertime, with RH higher than 75%. An ever-existing challenge for a ground source heat pump system is depleting the ground energy. Hence, it is particularly important to compensate for the extracted energy during the heating season by injecting heat into the lake during the greenhouse cooling season. Since in this study the heat pump is assumed to be off in summer, making use of the free cooling opportunity will compensate the extracted energy that occurs during the winter. In the case of the King Beaver Mine, it may not be absolutely necessary to return heat to the reservoir. However, this could be very important for a smaller water reservoir, such as a large underground cistern.

## Results and discussion

By completing the first three steps of the graphical Pinch Analysis method (data extraction, data processing, and problem setup), and obtaining the possible hot and cold streams, it is possible to find appropriate stream pairs in each time step in every typical day, by following the golden rules of the pinch method (Smith, 2005). Before that, it is worth recalling Heating/cooling load- Data Processing (Finding typical days) section, where the Typical Days (TD) were obtained by a clustering approach. Summarizing, the resulting six clusters of typical days can be categorized according to the heating/cooling demand: Heating only, Heating/Cooling, or Cooling only.

### Energy targeting: Dynamic pinch analysis

**Figure 12.**
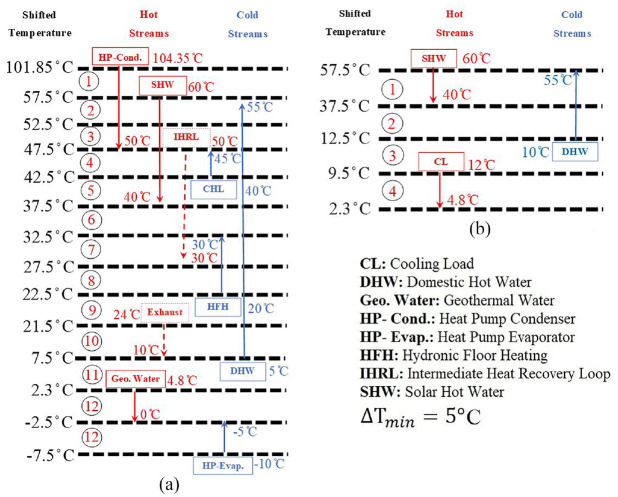
The streams in shifted temperature intervals: (a) streams for TD1 in winter and (b) streams for TD6 in summer.

Figure 12 presents the streams required to satisfy the energy demand of the case study greenhouse for typical day TD1 during the winter (when the greenhouse requires heating only), on the left side, and for TD6 in the summertime (when the greenhouse requires cooling only), on the right side. It should be noted that, except for the heat pump, the hot and cold streams have time dependent trends in each TD. Hence, in this study a dynamic PA was applied. As can be seen in Figure 12(b), for the hottest season, the heat pump system will be kept off, and only the free cooling option (geothermal water) will serve as cooler/ dehumidifier. It should be highlighted that not all streams shown in Figure 12 participate in every time slice for that particular typical day. By choosing this operating mode, the operational cost for the greenhouse is very low, while its products are preserved from outside pests. In this study each TD is divided into 24 portions, each defined as a Time Slice (TS) of 1 h, and PA is implemented (Hosseinnia and Sorin, 2022). Equation (2) determines the average Time Slice heat capacity (kW/K) for each stream.

(2) $${\bar{\mathrm{CP}}}_{\mathrm{TSt}}=\frac{\int_{t}^{t+\mathrm{TS}}\mathrm{CP}\left(t\right)\mathrm{dt}}{\mathrm{TS}}\mathrm{for}\;\mathrm{small}\;\mathrm{TS}\;{\bar{\mathrm{CP}}}_{\mathrm{TSt}}=\mathrm{CP}(t)$$

**Figure 13.**
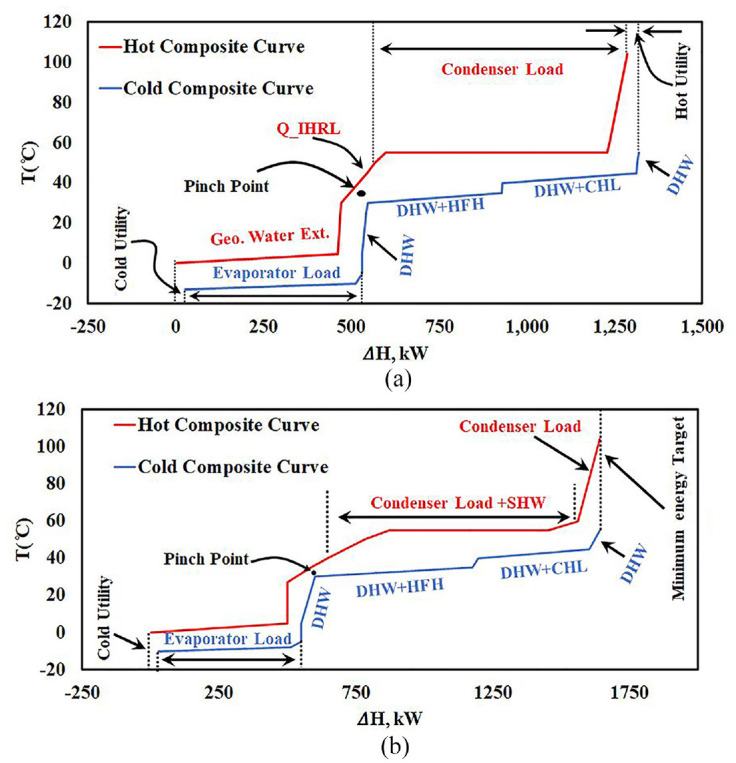
Composite curves for TD1 at different times of the day: (a) composite curves for streams at midnight (b) composite curves for streams at 15:00.

Figure 13(a) and (b) show the composite curves for different streams for the case study greenhouse at midnight for TD1 (Heating only demand) and TD6 (Cooling only demand), respectively. With respect to Figure 13(a), it should be highlighted that at midnight there is no SHW available, and the heating demand is greater than what the condenser duty can provide. Hence, to meet the heating demand, an Intermediate Heat Recovery Loop (IHRL) transfers hot water from the TES to the coil to satisfy design parameters. This design detail will be covered in the system design section of a future publication. Figure 13(b) shows the composite curves for TD1 in at 15:00, when all streams are available. As shown in Figure 13(b), minimum energy consumption and maximum heat recovery potential are achieved, while a small amount of cold utility is required, which is negligible.

**Figure 14.**
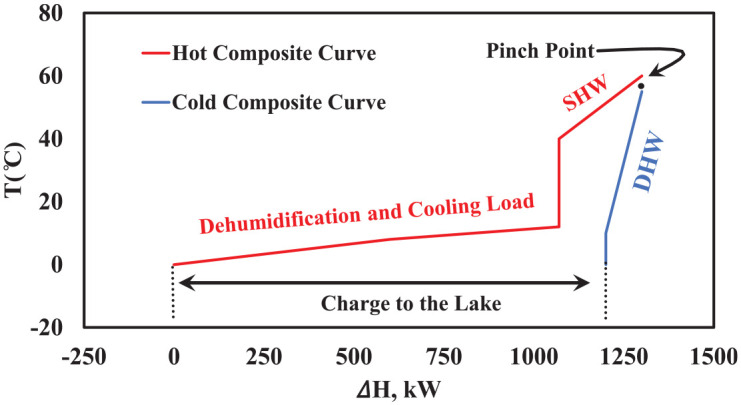
Composite curves for TD6 at noon (12:00 pm).

Figure 14 shows composite curves for the case study greenhouse in TD6 at noon, in summer, when only cooling load is present. As presented in Figure 12(b), on this typical day only three streams participate, two hot (SHW and CL) and one cold (DHW). As explained before, by taking advantage of the free cooling opportunity, by absorbing heat from the greenhouse, the open pit lake compensates for the energy extracted during the winter and reduces the summer energy cost by allowing the heat pump to be turned off. Moreover, sizing a heat pump for a greenhouse which can meet the energy demand in summer and winter is not logical, since the summer cooling load is almost three times higher than the winter heating load, and this for a brief period.

No hot utility is required in Figure 14, which shows a typical summer day at 12:00 p.m. However, during nighttime when there is no solar irradiance, some hot utility is required. To provide a more robust and dependable energy system, an additional TES will be added to the SHW, which will be called the Auxiliary Thermal Energy Storage (ATES) in this study. This storage tank has the same role of the TES for the heat pump, but it will be connected to the solar system. The size of the ATES can be calculated in the same way that the TES was sized.

**Figure 15.**
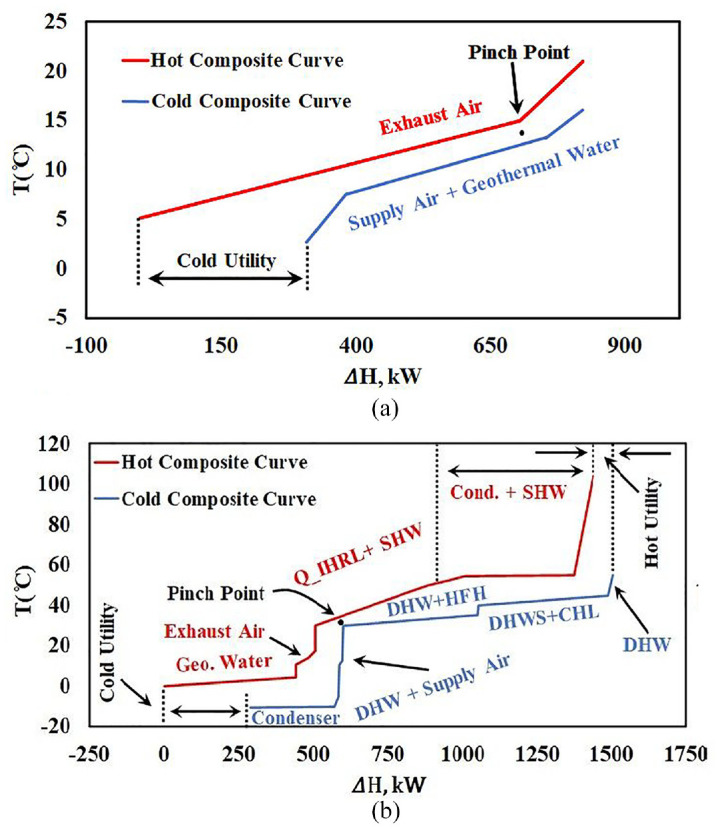
Composite curves for TD4 at different times of day: (a) 11:00 and (b) 18:00.

The remaining operating mode, which is the most sophisticated one, requires designing an energy system for TD3 and TD4, when the greenhouse has heating and cooling loads in a single day. For example, the greenhouse might need heating during the night and cooling/dehumidification during noon and afternoon or even night. For this operating mode, the considered energy system will include an AHU with the greenhouse. In this operating mode, the heat pump cannot work under constant load (smaller size in comparison with winter season) for 24 h a day. Instead, The HP thus has On/Off status during the day. However, the HP can still store excess energy in the TES, and when cooling/dehumidification is needed it changes its status to Off, and the AHU will remove heat and moisture from the inside air to either geothermal water or to the exterior air. Also, it can be used to preheat refrigerant in the evaporator, as explained in Ghaderi et al. (2023). Figure 15 shows the composite curves for this situation in TD4, when the greenhouse needs heating during the night and cooling during the day. Figure 15(a) presents the function of the AHU without considering any other hot and cold streams. In fact, the participating streams are supply air, exhaust air, and geothermal water. In contrast, Figure 15(b) presents all the greenhouse hot and cold streams for TD4. As shown in Figure 15(a), the exhaust air carries a huge amount of thermal energy, mainly because of its significant water content. The high slope of the hot composite curve before the dew point (right hand side of pinch point) is because of moisture removal from exhaust air. At the time that is presented in Figure 15(a), the outside temperature is lower than the inside air temperature. Hence, part of exhaust air energy can be used to preheat the incoming supply air to the greenhouse, and the rest can go to regenerate the open pit lake. Figure 15(b) shows the composite curves for 18:00 h, when almost all streams are present, and the AHU is still active.

### Heat pump and TES capacity

A heat pump can be sized based on its evaporator or condenser load, with different approaches for each. It is known that designing a heat pump system for the hottest or coldest time of year can lead to over-sizing or under-sizing. In Canada, NRCAN proposes a design temperature for different provinces. This temperature can cover more than 99% of days of a year. However, when it comes to greenhouse design temperature, this method will not work for two reasons. First, a greenhouse cannot rely on the thermal inertia of its structure, and second, plants cannot survive extreme conditions, even for short periods of time.

**Figure 16.**
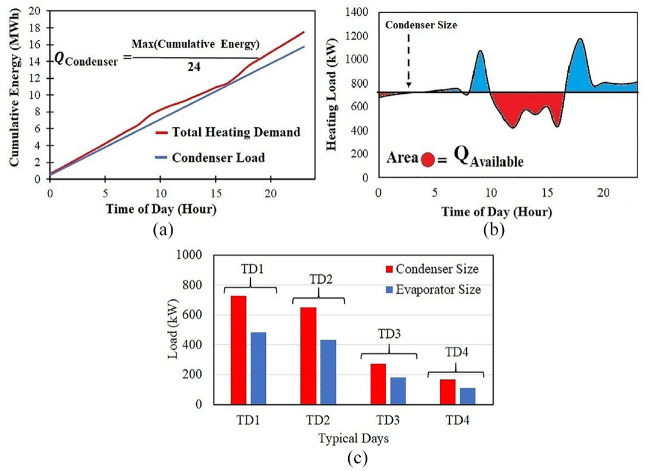
Time pinch analysis for optimum condenser load calculation: (a) condenser sizing for TD1, (b) TES sizing for TD1, and (c) HP equipment size for different TDs.

Hosseinnia and Sorin (2021) introduced the time pinch analysis approach to size a heat pump condenser for constant load. Also, the designed HP should be connected to a TES to supply or store required/surplus hot water requirements for heating purposes throughout a 24-h period. Figure 16(a) shows the method to size the HP’s condenser, which is proposed to integrate with the open pit lake of King Beaver mine. As illustrated in Figure 16(a), the condenser load is equal to the slope of the tangent line (blue line) to the cumulative energy profile. After sizing the HP, it is possible to size the TES that should be integrated with HP. The blue area in Figure 16(b) represents the thermal output from the TES for 1 day. The total area of the red region in Figure 16(b) equals the amount of energy that can be stored in the TES in 1 day. By using this value and equation (3), the TES volume is calculated. In equation (3), ρ , $C_{p}$ , and ΔT are the density and specific heat capacity of water, and the temperature difference of the stratified TES, respectively. The value of ΔT , which is a TES design parameter, should be set for each TD, and can be varied from 10°C to 35°C (Hosseinnia and Sorin, 2021). Hosseinnia and Sorin (2022) considered this value as 35°C in their research, while in this study it is set to 25°C. Figure 16(c) shows the HP’s equipment size (condenser and evaporator) for TDs in Heating only demand (TD1 and TD2) and Heating/Cooling demand (TD3 and TD4) operating modes.

(3) $$V_{\mathrm{TES}}=\frac{Q_{\mathrm{available}}}{\rho C_{p}\Delta T}$$

For the pure heating demand typical day (TD1), which covers 12% of the entire year (Figure 6), the condenser size is calculated as 728 kW, while the maximum heating load for this typical day is 1170 kW, which shows around a 40% reduction in heat pump size when a TES is used. Also, the size of the TES that should be connected to the heat pump is estimated at 14.3 $m^{3}$ . During the typical day, when heating demand is less than HP condenser thermal output, hot water will be stored in the TES. When the heating demand exceeds available condenser thermal output, the heating system will use the stored hot water. Please be aware that determining the suitable size for the heat pump based on typical days requires an economic evaluation, which is beyond the scope of this paper.

### The importance of the AHU

**Figure 17.**
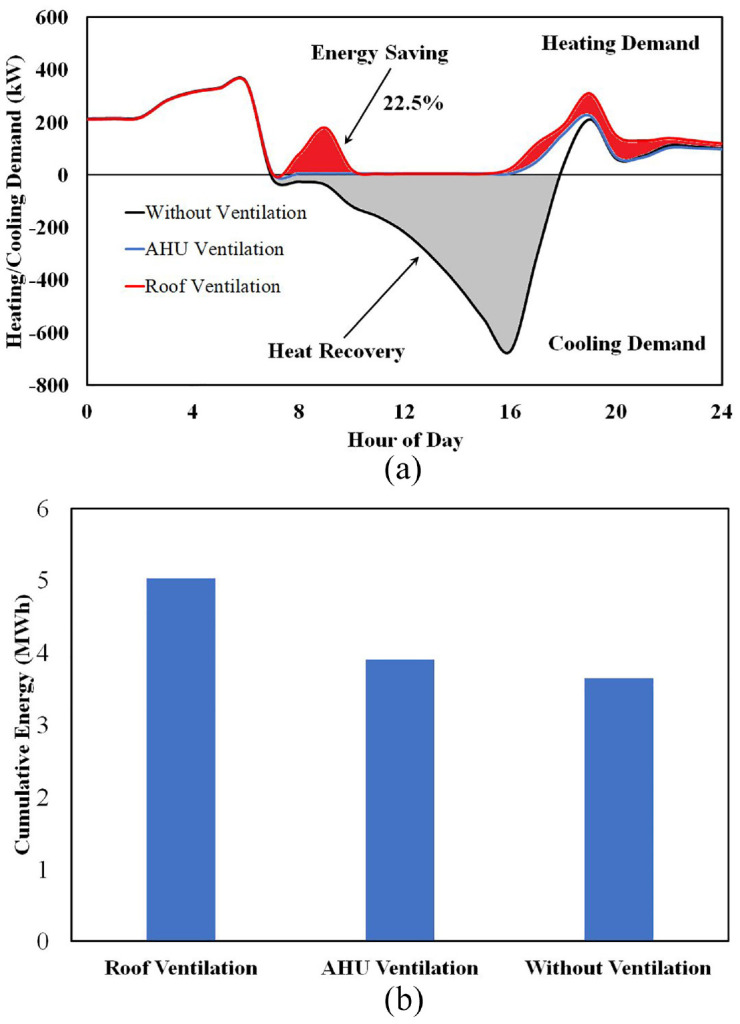
(a) Heating and cooling demand in TD4. Positive and negative values represent heating and cooling demand, respectively and (b) cumulative heating energy with different ventilation strategies.

In Energy targeting: dynamic pinch analysis section, it was investigated that for TD3 and TD4, when the case study greenhouse needs both heating and cooling, that instead of using the roof ventilation system, it is better to use the AHU to cool down the greenhouse. The energy removed from the greenhouse would be sent to the open pit lake (charging the geothermal resource), partially compensating for the extraction of energy during the winter. However, the quantity of energy that can be saved remained unknown. To quantify this value, Figure 17 shows and compares the heating demand of the St. Elie greenhouse at TD4, evaluating three different ventilation strategies, namely Roof Ventilation (RV), Air Handling Unit Ventilation (AHUV), and No Ventilation (NV). As suggested by its name, in the AHUV strategy, when cooling demand is necessary, the AHU provides the cooling duty either from geothermal water or from evaporator refrigerant. In the RV strategy, the hot and humid air will be exhausted through roof ventilation. As can be seen in Figure 16, the RV strategy includes unnecessary heating in TD4.

Investigating Figure 17(a) clarifies two points: first, the amount of energy that can be charged to the geothermal water (gray area), and second, the amount of energy required in heating load. The grey area shows the amount of cooling load, which means the greenhouse air with temperature and/or humidity greater than the setpoint must to be exhausted. This significant quantity of energy can be stored in the open pit lake to compensate the extracted energy in winter. Also, by comparing AHU Ventilation (blue) and Roof Ventilation (red) curves, and considering Figure 17(b), which shows the cumulative heating loads for the greenhouse based on different ventilation strategies, it is apparent that the AHU ventilation heating demand is significantly less than the heating demand when roof ventilation is active. This indicates that integrating an AHU within the greenhouse energy system reduces the heating demand by as much as 22.5%.

**Figure 18.**
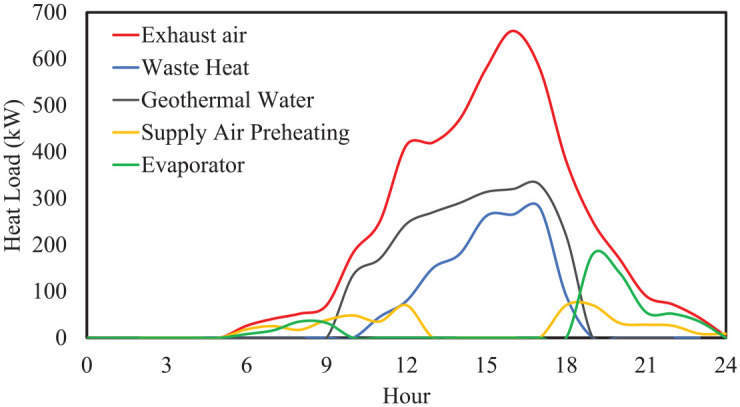
Exhaust air heat breakdown for TD4.

Pinch analysis can determine where to direct this amount of recovered heat, whether to transfer it to geothermal water or to preheat the refrigerant (evaporator). Figure 18 shows the contribution of different streams to the exhaust air for TD4. As depicted, at noon (12:00 pm) exhaust air can deliver a significant amount of heat to cold streams, and this value peaks at 3 pm. Also, since the afternoon outside temperature is sufficiently hot, it is not necessary to transfer energy from the exhaust air to preheat the incoming supply air. As a consequence, there is a great potential to thermally recharge the open pit lake water, or even to store this amount of energy in a low enthalpy TES to supply the evaporator when there is nightime greenhouse heating demand. In the case of TD4, nearly half of the heat typically extracted by a traditional ventilation system can be reclaimed through efficient integration of waste heat from exhaust air. Considering the example of the recoverable heat in Figure 18, 16% could be used to preheat incoming greenhouse air, 14% could be allocated to suppling energy to the evaporator, and 32% could be stored in the lake water. This figure shows possible connections between streams, and also gives some idea of the relative sizes of the related heat exchangers.

### Conclusion

Reactivating abandoned mines by extracting geothermal energy from them to meet the energy demand of greenhouses, in cold climate countries like Canada, is a promising proposition. This paper introduced a systematic method based on dynamic PA to determine the potential of the exhaust air from roof ventilation and renewable energy resources (solar collectors and geothermal energy from accumulated water in abandoned mines) to be integrated into greenhouses in cold climates. An important contribution of this study is the originality in the methodology enabling PA to be used with a dynamic set of streams. This study focused on targeting minimum energy consumption, maximizing heat recovery, and recharging the low temperature geothermal resources. The considered system hypothetically combines the geothermal water resources of an abandoned mine, namely King Beaver Mine, with the heating/cooling demands of a commercial greenhouse, les Serres St-Élie. A four-step procedure for conceptual design of the considered energy system was followed. In the first step, which is called data extraction, the values for all energy sources and sinks (solar energy, HP including evaporator and condenser, DHW, HFH, and TES) are calculated, and converted into hot and cold streams. Since most of these energy sources and sinks are time-dependent (dynamic), to reduce calculation time, in the second step, two clustering methods (K-Means and K-Medoids) were employed to find six typical days (TDs) that represent one complete year. Then, these TDs were regrouped into three demand categories: Heating only, Heating/Cooling, Cooling only. In the third step, which is called problem setup, the composite curves, and grid diagrams are drawn. In the final step, called the Dynamic Pinch Approach (DPA), the possible number of heat exchangers and the size of the heat pump and TES are determined, and the amount of heat recovery for each TD is calculated. Calculations showed more than a 40% reduction in the HP system’s equipment size (condenser and evaporator). Also, it was demonstrated that it is possible to integrate a HP with TES, first to guarantee that the HP works under constant load (preventing on/off cycles and thus lowering maintenance cost), and second to resolve the issues related to the dynamic daily patterns of DHW, HFH and CL requirements. AHU integration with the greenhouse energy system also showed a significant advantage in terms of saving energy, reducing greenhouse energy consumption in TD4 by 22.5%.

### Future work

Dynamic Pinch Approach (DPA) has shown its potential to provide the preliminary conceptual design of an energy system with a specific contribution of different sources of energy. However, a complementary study is needed to find the economically viable design and optimal system size, while satisfying as much as possible the targets set by PA. That study will consider various design scenarios and make the selection of the most practical HEN topology, accounting for energy, practical and economic considerations (i.e. thermo-economic analysis). At the conclusion of these studies, it will be possible to develop a software tool for designing a large-scale, multi-zone greenhouse that is integrated with different energy resources, whether fossil fuels or renewable energies.

## Appendix

### Notation

$A_{c}$ Solar collector area ( $m^{2}$ )

$A_{\mathrm{roof}}$ Roof area $\left(m^{2}\right)$

*CP* Mass Flow rate × cp, heat capacity rate (J $K^{-1}S^{-1}$ )

$C_{p}$ Specific heat capacity (J $kg^{-1}K^{-1}$ )

G Solar irradiance (W/ $m^{2}$ )

*H* Enthalpy (kW)

$Q_{\mathrm{SHW}}$ Solar hot water duty (W)

$Q_{\mathrm{Available}}$ Energy to be stored (J)

*T* Temperature (°C)

$T_{c}$ Average collector temperature (°C)

$T_{\mathrm{out}}$ Ambient temperature (°C)

$T_{\mathrm{in}}$ City water temperature (°C)

ΔT Temperature difference (°C)

ΔH Enthalpy difference (kW)

$V_{\mathrm{TES}}$ Thermal energy storage volume ( $m^{3}$ )

### Greek letters

$\eta_{0}$ Nominal solar collector efficiency

$a_{1},a_{2}$ Solar collector constants

ρ Density (kg $m^{-3})$

z Solar collector slope (°)

### Acronyms

AHUV Air handling unit ventilation

ATES Auxiliary thermal energy storage

AHU Air handling unit

CCs Composite curves

CL Cooling load

COP Coefficient of performance

DHW Domestic hot water

DPA Dynamic pinch approach

GSHP Ground source heat pump

GWP Global warming potential

HP-Cond. Condenser of heat pump

HP-Evap. Evaporator of heat pump

HEX Heat exchanger

HEN Heat exchanger network

HFH Hydronic floor heating

HP Heat pump

HTF Heat transfer fluid

HU Hot utility

HRV Heat recovery ventilation

IHRL Intermediate heat recovery loop

MHR Maximum heat recovery

NV No ventilation

PA Pinch approach

RV Roof ventilation

s Entropy

TD Typical day

TES Thermal energy storage

TS Time slice
